# ‘Skin in the Game:’ Social Goals Implementation Intentions Increase Intentions to Comply with COVID-19 Preventive Measures

**DOI:** 10.5334/irsp.802

**Published:** 2024-05-14

**Authors:** Jais Adam-Troian, Sylvain Delouvée, Eric Bonetto

**Affiliations:** 1 School of Psychology, Heriot-Watt University, Dubai Campus, United Arab Emirates; 2 LP3C, Rennes 2 University, France; 3 PSYCLE, LPS, InCIAM, Aix-Marseille University, France

**Keywords:** implementation intentions, social goals, COVID-19, preventive behavior, compliance

## Abstract

Despite the proven effectiveness of COVID-19 preventive measures (social distancing, frequent hand washing, vaccination, etc.), these remain inoperative if individuals do not adopt them. In this research, we sought to investigate the effectiveness of a novel type of intervention to foster compliance with COVID-19 preventive measures. Drawing upon the model of action phases and recent evidence linking social motives to compliance with recommendations from health authorities, we extended implementation intentions to the realm of social goals (Social Goals Implementation Intentions, or SGII). In a first study in France (*N* = 161), we show that a brief writing task requiring participants to implement a future hypothetical encounter with a close one at risk for severe symptoms of COVID-19 increased compliance intentions by 6.38% 95%*CI*[1.56, 11.24], *d* = .42, relative to a deliberation-only control condition. No moderating role of conspiracy beliefs and mentality was found in exploratory analyses. These results were exactly replicated in a pre-registered study conducted among US participants (*N* = 223), where the increase caused by SGII was 7.18% 95%*CI*[2.10, 12.27], *d* = .40. Vaccine intentions were not affected in both countries. Taken together, our results suggest that SGII is a viable theoretical mechanism to design and implement health behavior change interventions. Generating a sense of ‘skin in the game’ may be more effective to bypass irrational beliefs and foster greater adherence to evidence-based health recommendations.

‘What matters isn’t what a person has or doesn’t have; it is what he or she is afraid of losing’.

Nassim Nicholas Taleb, 2017, *Skin in the Game: Hidden Asymmetries in Daily Life*

## Introduction

As of March 10, 2023, 6.88 million individuals had died due to the COVID-19 pandemic worldwide.^1^ To curb the pandemic, prevent cases, and save lives, a number of pharmaceutical and social-behavioral tools (van Bavel et al., 2020) have been developed, tested, and implemented by governments across the globe. As part of the public health toolbox to fight the COVID-19 pandemic, effective vaccines have been developed and distributed to inoculate citizens against the virus (Knoll & Wonodi, 2021; Li et al., 2021). Globally, the use of facemasks to prevent contamination in the first place was generalized in public spaces (Howard et al., 2021). Likewise, public health authorities in various countries have recommended and enforced social distancing rules to limit airborne transmission (sometimes with stay-at-home orders and lockdowns) (Fazio et al., 2021). Other measures like contact-tracing and frequent hand sanitization were also encouraged as effective preventive measures (Fetzer & Graeber, 2021; Mukherjee et al., 2021).

Despite the proven effectiveness of these various pharmaceutical, technological, and behavioral techniques in limiting the spread and severity of COVID-19 cases, they remain useless if individuals do not adopt them. Currently, the main barrier to most COVID-19 preventive tools remains individual compliance with public health recommendations (Daoust et al., 2021). In some countries, groups of non-compliers with COVID-19 vaccination recommendations (e.g., US Republicans) were found to suffer from excess COVID-19 mortality rates (Morabia, 2023). Likewise, meta-analytical estimates suggest that individual enactment of protective measures (e.g., handwashing, mask wearing, social distancing) is associated with a reduced incidence of COVID-19 (Talic et al., 2021). In addition, research shows that countries where compliance with norms is enforced more strictly (i.e., ‘tight’ cultures) (Gelfand et al., 2011) tend to experience lower COVID-19 cases and deaths, even after accounting for a host of potential confounds (Gelfand et al., 2021). Thus, ensuring compliance with COVID-19 preventive measures remains crucial to contain the pandemic, especially in ‘looser’, more individualistic cultural contexts.

Focusing on the US and France, we test the effectiveness of a novel, brief psychological intervention aimed at increasing individuals’ intentions to comply with COVID-19 preventive measures: Social Goals Implementation Intentions (SGII). This approach combines insights from the action phase model of behavior change (Keller et al., 2020) with recent evidence showing that prosocial motives may underlie compliance with preventive measures (Lachowicz-Tabaczek & Kozłowska, 2021) to target social goal attainment directly: meeting with a friend or relative at high risk of mortality from COVID-19.

### Fostering Compliance: From Epistemic to Social Goals

In the domain of health behavior, interventions targeting attitudes typically yield medium-sized effects (Sheeran et al., 2016). More recently, experimental studies conducted in the context of the COVID-19 pandemic showed that support for social distancing can be increased by simply correcting misperceptions of the pandemic growth as linear and not exponential (Lammers et al., 2020). An overview of the work conducted to boost COVID-19 vaccination rates in France also shows that behavioral interventions, such as vaccine mandates, could impact their target without necessarily impacting vaccine hesitancy or health beliefs regarding vaccines (e.g., safety and effectiveness) (Ward et al., 2022).

An emerging line of research on preventive behavior in the context of the pandemic suggests that individuals do not merely act upon purely epistemic motives (e.g., seeking truthful information or acting upon information they believe is true). Rather, preventive health behavior seems to be substantially shaped by social motives. For instance, a strong sense of national identification positively relates to support for and compliance with COVID-19 preventive measures (in 67 countries) (Van Bavel et al., 2022). Likewise, psychosocial factors, among which social and subjective norms, constitute robust cross-cultural predictors of compliance with preventive measures (e.g., in France and Belgium) (Wollast et al., 2021; Schmitz et al., 2022). In line with these results, a series of studies conducted recently in France highlighted that feelings of closeness to family and national identification were both substantial, positive predictors of intentions to comply with preventive measures (Marinthe et al., 2022). The SGII draws upon these studies and the action-phase model of behavior change (Keller et al., 2020). It aims to set social goals in order to target behavioral intentions to engage with preventive measures directly.

### The Present Research: Social Goals Implementation Intentions

The model of action phases posits that two sequential processes lead to individual enactment of behavior: motivational (i.e., goal setting) and volitional (i.e., goal implementation). Hence, the model of action phases recommends different types of intervention be implemented depending on whether individuals are still at the goal-setting stage (pre-decisional phase) or have already moved to the SGII stage (post-decisional phase; Keller et al., 2020). Supported by decades of empirical research (Brandstätter et al., 2003; Gollwitzer, 2012; Puca & Schmalt, 2001; Keller et al., 2019), the most notorious application of the model has been implementation intentions.

Briefly put, implementation intentions are an intervention at the post-decisional phase that consist of specifying a plan to act upon one’s intentions by asking individuals to think about how, when, and where they should display the target behavior (Gollwitzer, 1999). By doing so, implementation intentions provide a roadmap for individuals, which allows them to anticipate and bypass potential barriers to behavior implementation. Implementation intentions have been proven effective in various domains (Gollwitzer & Sheeran, 2006), including health behaviors (Armitage, 2016; Robinson et al., 2019; Silva et al., 2018), and remain to this day one of the most effective tools to promote costly behaviors such as regular physical exercise (Milkman et al., 2021).

In the context of the COVID-19 pandemic, implementation intentions were tested by an interventional study, which demonstrated the effectiveness of these simple ‘if-then’ plan formulations in fostering greater compliance and preventive measures (Ahn et al., 2021). In this original study, Ahn et al. (2021) randomly allocated 197 participants to an implementation intervention or control group, measuring their adherence to the CDC social distancing guidelines every day for a week and then at a three-week follow-up. Goal commitment (i.e., an individual’s reported commitment to committing to the goal of social distancing in the near future) was also measured. Although encouraging, the innovative study from Ahn et al. (2021) was limited in two important ways. First, the outcome measure only pertained to social distancing behavior. Second, the intervention did not have a main effect. The effects were fully moderated by goal commitment, meaning that the intervention mostly fostered compliance among already committed individuals.

These limitations could be due to the operationalization of Ahn et al.’s (2021) goal-setting phase, which was to protect ‘yourself and others from COVID-19 by putting six feet of distance between yourself and people who don’t live in your household’. In fact, linking the goal to one specific behavior constrains the scope of the intervention to the recommended behavior. Also, protecting ‘oneself and others’ may be too abstract a goal, especially for individuals who may consider themselves or cognitively accessible ‘others’ not at-risk for various reasons (e.g., religiosity or young age). Moreover, the model of action phases predicts that individuals who do not believe in the effectiveness of preventive measures could experience an impeding discrepancy between set goals (protecting oneself or others) and recommended implementation (social distancing).

Interestingly, the model of action phases allows considering a variety of ‘set goals-implementation behavior’ pairs to determine goals to target in an intervention. If social distancing behavior is included in the set of behaviors that facilitate attaining the goal of protecting oneself or others against the virus, then, conversely, it is not true that the set of behaviors allowing one to achieve this goal is limited to social distancing only. Nor is it true that the set of goals that can be attained through implementing social distancing is limited to ‘protecting oneself and others’. This is where the evidence provided by Marinthe et al. (2022) on the importance of considering social motives comes into play. In this study, we propose to test the effectiveness of a modified implementation intentions procedure to include a goal-setting phase that includes more vivid social concerns for one’s relatives and friends at high mortality risk from COVID-19.

### Hypothesis

We argue that facing a real and vivid choice, which involves a huge cost if not properly made, most individuals would base their decisions on social (doing right) instead of epistemic (being right) motives. Thus, our procedure aimed to tap into powerful specific motives by setting a vivid social goal in lieu of an abstract and general motive. Hence, compared to a control condition, we hypothesized that implementing intentions to attain a social goal (visiting a friend or relative at high mortality risk from COVID-19) would increase intentions to comply with most COVID-19 preventive measures, including vaccination. We decided to test this hypothesis in our first study in France. The results of this study were then used to preregister a replication study in the US (https://osf.io/fmj26/?view_only=a2a38988879043ffb004d4a35df0e77d).

### Ethical statement

The studies were all conducted in accordance with the APA Code of Conduct (APA, 2017). We report all measures, manipulations, and exclusions in these studies. Supplementary materials, analyses, and all the data underlying our findings can be openly accessed and downloaded through the Open Science Framework platform at https://osf.io/fmj26/?view_only=a2a38988879043ffb004d4a35df0e77d.

## Study 1

### Method

#### Power Analyses & Participants

Prior to data collection, we conducted a power analysis with GPower (Faul et al., 2009) to determine the appropriate sample size for this first study. We decided to set the expected effect size to *d* = .65, following meta-analytical estimates of implementation intentions effects from Gollwitzer and Sheeran (2006). Setting target power at 90% with an α = .05 and using two-tail independent *t*-tests (to limit the risk of type I errors), analyses revealed that a minimum of 102 participants (51 per cell) was needed.

We decided to conduct the study among a convenience sample of French psychology undergraduates. A total of 166 undergraduate students took part in the study. Among those, 3% (n = 5) provided obviously fake answers to the writing tasks and, most importantly, did not provide a target for the manipulation (see FILTER variable in the dataset: https://osf.io/fmj26/?view_only=a2a38988879043ffb004d4a35df0e77d). These participants were filtered out for the analyses. Our final sample thus comprised 161 students (21.1% male, *M_age_* = 19.0; *SD* = 6.44; *N_intervention_* = 79; *N_control_* = 82), guaranteeing sufficient power to implement our tests. A sensitivity analysis revealed that this sample size allowed us to still detect effects of size *d* = .51 with similar specifications as those mentioned above.

#### Procedure

Students were informed that participation in this study was available in exchange for course credit during a social psychology lecture on social representations theory held by one of the authors at a French university in March 2021. The university had switched all its courses online. The study was an online survey experiment: the PI sent the link to a Qualtrics survey to all students’ emails via the university’s Moodle platform. The study was available for one week, and around 70% of the students enrolled in the lecture took part.

#### Study design

The study was designed along a straightforward one-factor, two-conditions (control, intervention) between-subjects plan. Participants were invited to fill out a first set of demographic and conspiracy beliefs measures. These were present only in the first study to explore the potential presence of interaction effects between the intervention and conspiracism (i.e., low commitment to complying with health measures). Participants were then randomly assigned to one of the two conditions. In both conditions, participants were informed that the study pertained to their ‘opinions about the recommended health behaviors to fight against COVID-19 in France’ and asked to carefully read a poster detailing ‘a list of behaviors recommended by the French Ministry of Health’ (following Ahn et al., 2021). This poster was taken from the ministry’s website and contained detailed recommendations on several measures, such as hand sanitization, wearing masks, vaccination, and social distancing. As part of the study’s cover story, they were then asked to rate the perceived effectiveness of recommendations to protect their own health, their relatives’, or that of French people in general.

Following this first common task, participants were then randomly assigned to one of two different tasks. In the intervention condition, they were invited to perform a three-part writing task consisting of a modified version of the traditional implementation intentions paradigm. A first brief question was designed to lower reactance by asking participants to rationalize their attitudes towards the preventive measures, removing any discrepancy or sense of threat prior to moving on to the goal-setting phase (‘How confident are you in your opinions regarding the effectiveness of these recommendations? Are you confident that your opinions are correct, factual and that others should have the same opinions as you?’; especially for participants with negative attitudes towards recommendations) (Kruglanski et al., 2018).

A social goal was then set by asking participants to ‘think of a close person at high risk of death from COVID-19. This person may be a family member, close friend or relative; someone you care about’ and briefly note who the person is (without giving away personal details, e.g. ‘my uncle’). Finally, intentions were implemented by asking participants to ‘imagine that you wanted to meet this person. How would you go about it? Would you meet them in their home, in a public place or online? Would you wear a mask or not? Would you respect at least one meter of social distance? Would you wait to be vaccinated?’ and to briefly explain how they would implement the encounter.

In the control condition, participants were asked to ‘think about your answers to the previous three questions. You will be asked questions about these recommendations again on the next page’. This provided us with an active control condition (deliberation, effective in generating more analytical and rational decision-making processes) (Bago et al., 2020; De Neys & Pennycook, 2019; Obrecht & Cheney, 2016). After being exposed to one or the other condition, all participants were invited to rate the perceived effectiveness of the recommended measures once more.

#### Measures

Prior to random allocation to one of the conditions, participants were asked to fill out the following measures:

##### Sociodemographic variables

Sex of participants, age, and political ideology (single-item, from 1= ‘far-left’ to 7= ‘far-right’; M = 3.06, *SD* = 1.10) were measured.

##### Additional measures

Before allocation to treatment groups, our survey also included three scales to measure COVID-19 news: skepticism, conspiracy mentality COVID-19, and conspiracy beliefs. We included them to assess their potential moderating roles in the intervention’s effect, but we could not reliably exploit these analyses due to a lack of power. Nonetheless, the full details of these underpowered analyses can be found in the ‘supplementary analyses’ folder of the OSF project page.

##### Dependent variable

After completing either the implementation intentions task or the control deliberation task, we finally asked participants to report their intentions to comply with 12 different COVID-19 preventive measures ‘if the opportunity arises in the future’ as per the ministry of health recommendations at the time of the study (e.g., ‘Wash your hands regularly for at least 20 seconds’; ‘Cough or sneeze into elbow or handkerchief’; slider scale from 0% ‘absolutely not’ to 100% ‘completely’, *M* = 72.10, *SD* = 15.70, *ω* = .77).

### Results

#### Randomization check

As can be seen in Table 1, there were no substantial between-group differences in terms of political ideology and demographics prior to allocation: .16 < *p*s < .40; .14 < *d*s < .18. We thus considered ourselves to have achieved successful randomization.

**Table 1:** Descriptive statistics of the two experiments and between-condition differences (Study 1, N = 161; Study 2, N = 240).


		CONTROL *M*(*SD*)	INTERVENTION *M*(*SD*)	*t*-VALUE (*df*)	*p*-VALUE	EFFECT SIZE (*d*)

*Study 1*						

*Demographics*	Age	19.59(8.96)	18.44(1.10)	1.12(159)	.26	.18

	%_male_	25.6	16.5	1.42(159)	.16	.22

	Ideology	3.14 (1.09)	2.99(1.12)	.85 (158)	.40	.14

*Outcome*	Compliance	69.01(16.29)	75.39(14.38)	2.60(155)	.01	.42**

*Study 2*						

*Demographics*	Age	45.01(17.14)	45.60(17.82)	.25 (221)	.81	.03

	%_male_	50.00	37.40	1.95(237)	.053	.25

	Ideology	4.04(1.77)	4.02(1.62)	.05(221)	.96	.01

*Outcome*	Compliance	78.49(19.93)	85.67(14.62)	2.78(205)	.006	.40**


*Note*. **p < .01.

#### Correlation analyses

Correlation between all measured variables can be seen in Table 2.

**Table 2:** Summary of correlation analyses between all measured variables (study 1, N = 161; study 2, N = 240).


*STUDY 1*		1	2	3	4

1.	Age	–			

2.	Gender	.19*	–		

3.	Pol. Ideology	–.09	.05	–	

4.	Compliance	–.16*	–.09	–.05	–

***STUDY 2***					

1.	Age	–			

2.	Gender	.15	–		

3.	Pol. Ideology	–.01	–.10	–	

4.	Compliance	.03	.05	.13	–


*Note*. Numbers represent Pearson correlation coefficients. *p < .05.

#### Intervention effectiveness

**Figure 1:**
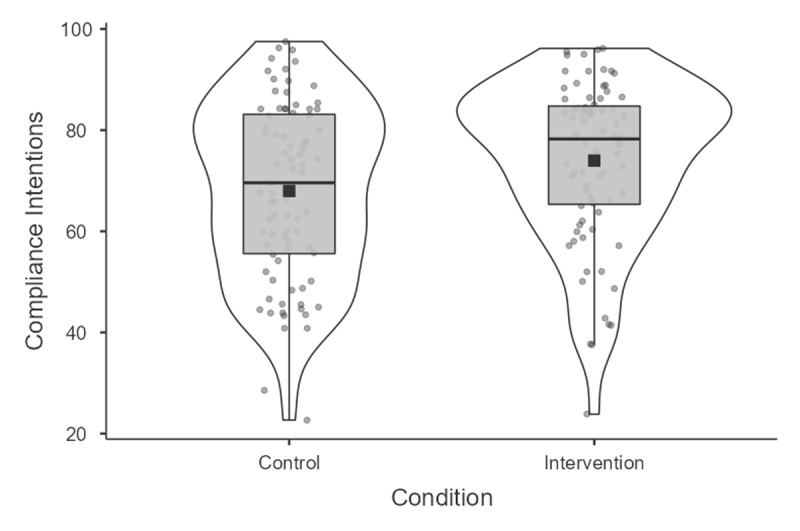
Compliance intentions across study 1’s conditions.

As hypothesized, the SGII had a substantial, positive effect on intentions to comply with preventive measures, *t*(155) = 2.60, *p* = .01. Intentions to comply were at a level of 69.01% in the control group relative to 75.39% in the intervention group, representing a difference of 6.38% 95%*CI*[1.56, 11.24], *d* = .42 (see Figure 1).

#### Exploratory analyses

Given that vaccination at the time of data collection was given in priority to older, at-risk individuals, we wished to explore whether results would remain the same with an index of compliance not including vaccination intentions. On a purely descriptive level, the intervention effect using this index seemed to be a bit stronger, *t*(155) = 2.83, *p* = .005, with intentions rising from 70.29% to 77.39% in the intervention group, or an increase of 7.09% 95%*CI*[2.51,12.06], *d* = .45. Also, the intervention did not seem to have affected vaccination intentions in any meaningful way, *t*(155) = .25, *p* = .83, *d* = .04. MANOVA results detailing the effect of the intervention on each individual behavioral intention item are also available in the ‘supplementary analyses’ section of the OSF project page.

### Discussion

This first study provided preliminary evidence for a potentially medium-sized effect of our social goal implementation intentions intervention. Nevertheless, the results of our intervention (the main effects of SGII) needed further corroboration. After analyzing data from study 1, we thus pre-registered a replication study (see https://osf.io/fmj26/?view_only=a2a38988879043ffb004d4a35df0e77d).

## Study 2

This replication study was conducted in the US for convenience reasons. Based on study 1’s results, we pre-registered the hypothesis that SGII would, again, lead to an increase in intentions to comply with COVID-19 preventive measures (H1a). This time, and contrary to study 1’s results, we expected that SGII effect would also extend to vaccination intentions (H1b). This was because we had no sufficient theoretical justification as to why SGII did not increase vaccination intentions specifically. However, there were reasons to suspect that this null effect could stem from the coercive take on COVID-19 vaccination in France, whereby the government boosted vaccine uptake rates by introducing vaccine mandates during the pandemic (i.e., the ceiling effect) (Ward et al., 2022). Thus, we had no reason to expect a null effect in the US context, marked by higher heterogeneity in vaccine policy and frequent overturns of vaccine mandate laws in some states (Mello et al., 2022).

In addition to providing for a replication of our study, the change in country would also test the cross-national generalizability of the intervention, at least within WEIRD contexts. This is because, despite both countries belonging to the WEIRD category, the two national contexts in France and the US do substantially differ from each other on several relevant dimensions. In terms of cultural values, for instance, the US context is still relatively more individualistic than France, much less long-term-oriented, and uncertainty-avoidant (Hofstede, 2011), which directly relate to the enactment of COVID-19 preventive behavior (Wang, 2021). As stated previously, France introduced vaccine mandates to access many public spaces (e.g., restaurants), while the application and enforcement of such mandates in the US were highly variable across states (Ward et al., 2022). Finally, the US is a much more politically polarized country than France (Dimock & Wike, 2020).

Again, we hypothesized that the compliance index would be positively impacted by the intervention. Given the importance of vaccination as a preventive measure against COVID-19, we also decided to analyze vaccination intentions separately. We predicted that, unlike in the context of restricted vaccination policy in France, these could be impacted by the intervention in the US, where vaccination was widespread by the time the study was conducted.

### Power Analyses & Participants

We preregistered a power analysis with GPower (Faul et al., 2009), setting the expected effect size at *d* = .45, following study 1’s exploratory results using the compliance index devoid of the vaccination item. Setting target power at 90% with an α = .05 and using two-tail independent *t*-tests (to limit the risk of type I errors), analyses revealed that a minimum of 210 participants (105 per cell) was needed.

Following our pre-registered exclusion criteria for filtering our ‘participants who obviously provided bogus/irrelevant answers to the exercise’ we systematically excluded participants who provided no target for the intervention, like in study 1. Thus, to achieve our target sample size, a total of 343 US citizens had to take part in the study, being reduced by 30.02%, leading to a sample of 240 individuals (21.1% male, *M_age_* = 45.20; *SD* = 17.40; *N_intervention_* = 100; *N_control_* = 140). Although this sample was larger than the 210 required, the condition imbalance due to exclusion rendered the intervention condition short of participants compared to our pre-registered requirements. Also, attrition led to a final *N_intervention_* = 85 and *N_control_* = 138, completing the outcome measure. Nonetheless, a post-hoc analysis revealed that this smaller and unequally distributed sample size allowed us to detect effects of size *d* = .45 under pre-registered parametrization with still 90.13% power.

### Procedure

Participants were sent a link to a Qualtrics survey via the recruitment platform Dynata, a US global online market research company. Data collection took place between January 12 and 19, 2022. The peak of 800,000 cases was then reached in the United States. Masks were mandatory in enclosed spaces and on public transport. In some states, masks were not required in enclosed spaces until the following month. The health situation was relatively comparable to that of study 1 in France, where masks were also compulsory in enclosed spaces and public transport without national lockdown but with a heavy strain on the hospital system.

### Study design

The study design was strictly identical to study 1’s, except that the COVID-19 recommendation poster was now that of the CDC to match the US context (https://osf.io/fmj26/?view_only=a2a38988879043ffb004d4a35df0e77d).

### Measures

Prior to random allocation to one of the conditions, participants were asked to fill in the same sociodemographic variables as in study 1, including political ideology (*M* = 4.03, *SD* = 1.71). After allocation, participants were invited to report their intentions to comply with COVID-19 preventive measures using the same items as those from study 1 (*M* = 81.27, *SD* = 18.36, *ω* = .94).

## Results

### Randomization check

As can be seen in Table 1, there were no substantial differences between groups in terms of political ideology and age, but groups did differ in terms of gender, *t*(237) = 1.95, *p* = .053, *d* = .25. With 50% men in the control condition versus 37.40% in the intervention group, we concluded that random allocation might have been compromised due to a gender bias in compliance with instructions on the writing tasks. Hence, we decided to perform additional robustness tests on our results with gender as a covariate (ANCOVA) in addition to the t-tests we had pre-registered initially.

### Correlation analyses

Correlation between all measured variables can be seen in Table 2.

### Intervention effectiveness

**Figure 2:**
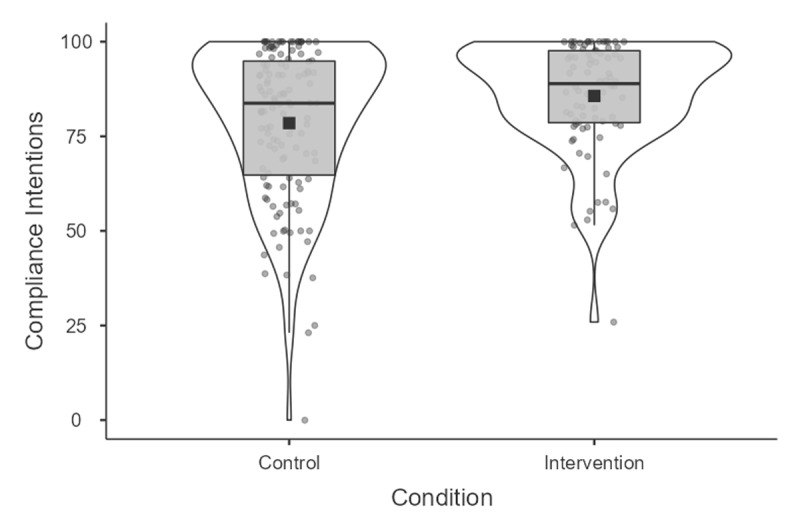
Compliance Intentions across study 2’s conditions.

Replicating study 1’s results and in line with our pre-registered first hypothesis, the SGII was found to exert a positive effect on intentions to comply with preventive measures, *t*(205) = 2.78, *p* = .006. Intentions were 78.49% and 85.67% in the intervention group, representing a difference of 7.18% 95%*CI*[2.10, 12.27], *d* = .40 (see also Figure 2). Again, and on a descriptive level, the intervention effect using the index without vaccination appeared as if it were slightly stronger, *t*(207) = 2.97, *p* = .003, with intentions to comply at 78.32% in the control group compared to 86.05% in the intervention group, or a difference of 7.73% 95%*CI*[2.60, 12.87], *d* = .42. Replicating study 1’s results, but contrary to our second pre-registered hypothesis, the intervention had once again no meaningful effect on vaccination intentions *t*(218) = .51, *p* = .61 *d* = .07. Although not pre-registered, MANOVA results detailing the effect of the intervention on each individual behavioral intention item are also available in the ‘supplementary analyses’ section of the OSF project page.

### Robustness checks

ANCOVAs were run with gender as a covariate and demonstrated that the intervention’s effect was robust for the compliance index with, *F*(1, 203) = 6.54, *p* = .011, *η*^2^= .03 or without the vaccination item, *F*(1, 205) = 7.58, *p* = .006, *η*^2^ = .04. Likewise, the introduction of gender as a covariate did not affect the intervention’s effect on vaccination intentions, *F*(1, 216) = .27, *p* = .60, *η*^2^= .00.

### Discussion

This second study allowed us to replicate the effects observed in France in study 1. Although our pre-registered hypothesis regarding vaccination intentions was rejected, the results were again very similar to those of study 1. Moreover, intervention’s absolute effect size in the US was an increase of 7.18% 95%*CI*[2.10, 12.27], in compliance intentions, which was close to that obtained in France, which was 6.38% 95%*CI*[1.56, 11.24]. Descriptively, the relative effect size in the US (*d* = .40) also seemed of similar magnitude to that obtained in France (*d* = .42). An important limitation remained: our participants’ gender was unequally distributed across our conditions. However, robustness tests showed that gender was not a likely confound.

## General Discussion

Across two studies in the US and in France, we obtained a consistent, medium-sized effect of our SGII intervention on compliance intentions with COVID-19 preventive measures, relative to an active deliberation control condition. The effect of SGII was in line with that obtained by Ahn et al. (2021), but it extended their findings in two important ways. First, we were able to observe a main effect of SGII, sweeping across participants in the studies. Second, the SGII procedure was able to foster increased compliance over a range of preventive measures (not limited to social distancing), with the notable exception of vaccination.

After conducting study 1, we hypothesized that failure to obtain an effect on vaccination intentions was due to contextual factors (the vaccination policy of reserving the limited supply of doses to older citizens at that time), which led us to pre-register an effect in the US. Study 2 invalidated this hypothesis, which raises interesting questions regarding vaccination intentions. The resistance of vaccination to social goal implementation intentions may be due to several elements that could be investigated in further research, such as greater invasiveness of the procedure (fear of injection) (Freeman et al., 2021) or a more complex procedure to follow, which could impede goal implementation (i.e., booking an appointment, locating, and going to a vaccine center) (Bieleke et al., 2021).

Theoretically speaking, the present results constitute the first evidence for considering three innovations in implementation intentions. First, our approach highlights how targeting the goal-setting phase and not only the implementation phase (Gollwitzer, 1999) can help bypass issues of reactance, resistance, or lower prior commitment to the goal itself.

Second, SGII demonstrates the relevance of the principle of equifinality (Kruglanski et al., 2018) in determining how goal implementation and target behaviors can be linked. Because several behaviors may sometimes equally serve to implement the same goal, targeting a goal broad enough to include several equifinal behaviors may prove effective in fostering compliance with more than one behavior in a single intervention.

Third, our studies suggest that considering social goals when implementing intentions could prove very effective. Here, setting the social goal ‘not to cause the potential death of a close one’ enabled the implementation of a range of preventive behaviors. Crossing insights from social influence research showing that conformity, consistency, or prestige are strongly influential factors in shaping behavior (Cialdini & Goldstein, 2004; Schultz et al., 2018) with those of the action phase of behavior change could help generate more powerful interventions.

### Limitations

Before concluding this series of studies and their overall implications, we wish to mention a few caveats that may impose constraints on the interpretation of our results. First and foremost, study 1 was conducted among a student population, and more specifically on a predominantly female student population (i.e., psychology undergraduates). Study 2 drew on a sample of the US population at large, but the sample was still comprised of a majority of women (78.9%). Although there is no specific evidence indicating that implementation intentions effects do not generalize across genders, caution is warranted with regards to potential gender differences. Because both the US and France are culturally close, we also advise that our results should not be generalized outside of the so-called WEIRD (Henrich et al., 2010).

Beyond these generalizability issues, we also wished to emphasize limitations due to the nature of our outcome variable. In fact, both studies made us aware of intentions to comply with health recommendations and not actual compliance behavior. Although behavioral intentions and corresponding behaviors are substantially linked, research shows that the size of this association is typically small to medium (Sheeran & Webb, 2016). Due to this intention-behavior gap, we also recommend that replication studies be conducted to determine the actual effect size of SGII interventions on actual behavioral outcomes.

Also, we did not compare our SGII intervention to a traditional implementation intentions intervention condition. Hence, this study does not provide evidence that social goals alone could be responsible for the observed effects. As such, the present research could be considered a phase I type of trial, where treatment effects are compared relative to a placebo condition (and not a treatment-as-usual group; that would be non-social goals implementation intentions). Still, our results differ substantially from those of Ahn et al. (2021), who used a non-social goals implementation intentions procedure and found no main effect of the intervention. In that sense, our study shows that SGII as an intervention holds potential for behavioral intention change, although we do not know if this is due to social goals planning exclusively.

Finally, we must also note an important peculiarity pertaining to study 2. In this US sample, the rate of non-compliant participants was 28.3%, almost ten times that among our French sample (3.01%). Although many factors affect compliance rates, the most parsimonious and likely explanation probably resides in the differences in data collection procedures across studies. While non-compliance includes but is not limited to dropouts, methodological research on dropout rates may be useful to interpret this difference in participants’ engagement across studies. While study 1 was conducted through a computerized survey disseminated among undergraduate students, which tends to produce dropouts of 10% or less under a survey length of 177 items (Hoerger, 2010), study 2 was conducted on an online platform (Dynata), which was found to have average dropout rates of 21.3% (within the range of study 2’s 28.3%) (Peer et al., 2022).

## Conclusion

Within the respective limitations of each study, these results show how a broader and social-goal oriented approach to action phases, including the goal-setting stage, could help design implementation intentions interventions to change multiple behaviors at once. Besides theoretical aspects, there is potential for further studies to maximize the applied benefits of our research endeavor. We recommend that future experimental tests expand on our intervention by using dismantling designs to pinpoint which part (or combined parts) of the procedure is necessary and sufficient to trigger change, if possible, using behavioral measures (Papa & Follette, 2015). More could also be done to include follow-up measures and assess whether the effects hold over sustained periods of time. Overall, we believe that social goals implementation intentions are a promising avenue for behavior change interventions.

## Data Accessibility Statement

Data underlying our findings are openly available at https://osf.io/fmj26/?view_only=a2a38988879043ffb004d4a35df0e77d.

## Footnotes

Source: John Hopkins Coronavirus Resource Center, available at https://coronavirus.jhu.edu/map.html.

## References

[1] Ahn, J. N., Hu, D., & Vega, M. (2021). Changing pace: Using implementation intentions to enhance social distancing behavior during the COVID-19 pandemic. Journal of Experimental Psychology: Applied, 27(4), 762–772. DOI: 10.1037/xap0000385 34424021

[2] American Psychological Association. (2017). Ethical principles of psychologists and code of conduct. Retrieved from https://www.lsbep.org/wp-content/uploads/APA-Ethical-Principles-of-Psychologists.pdf. Accessed February 28, 2022.

[3] Armitage, C. J. (2016). Evidence that implementation intentions can overcome the effects of smoking habits. Health Psychology, 35(9), 935–943. DOI: 10.1037/hea0000344 27054302

[4] Bago, B., Rand, D. G., & Pennycook, G. (2020). Fake news, fast and slow: Deliberation reduces belief in false (but not true) news headlines. Journal of experimental psychology: general, 149(8), 1608–1613. DOI: 10.1037/xge0000729 31916834

[5] Bieleke, M., Keller, L., & Gollwitzer, P. M. (2021). If-then planning. European Review of Social Psychology, 32(1), 88–122. DOI: 10.1080/10463283.2020.1808936

[6] Brandstätter, V., Heimbeck, D., Malzacher, J., & Frese, M. (2003). Goals need implementation intentions: The model of action phases tested in the applied setting of continuing education. European Journal of Work and Organizational Psychology, 12(1), 37–59. DOI: 10.1080/13594320344000011

[7] Cialdini, R. B., & Goldstein, N. J. (2004). Social influence: Compliance and conformity. Annual Review of Psychology, 55, 591–621. DOI: 10.1146/annurev.psych.55.090902.142015 14744228

[8] Daoust, J. F., Nadeau, R., Dassonneville, R., Lachapelle, E., Bélanger, É., Savoie, J., & van der Linden, C. (2021). How to survey citizens’ compliance with COVID-19 public health measures: Evidence from three survey experiments. Journal of Experimental Political Science, 8(3), 310–317. DOI: 10.1017/XPS.2020.25

[9] De Neys, W., & Pennycook, G. (2019). Logic, fast and slow: Advances in dual-process theorizing. Current Directions in Psychological Science, 28(5), 503–509. DOI: 10.1177/0963721419855658

[10] Dimock, M., & Wike, R. (2020). America is exceptional in the nature of its political divide. Retrieved from https://www.pewresearch.org/short-reads/2020/11/13/america-is-exceptional-in-the-nature-of-its-political-divide/. Accessed October 13, 2022.

[11] Faul, F., Erdfelder, E., Buchner, A., & Lang, A. G. (2009). Statistical power analyses using G*Power 3.1: Tests for correlation and regression analyses. Behavior research methods, 41, 1149–1160. DOI: 10.3758/BRM.41.4.1149 19897823

[12] Fazio, R. H., Ruisch, B. C., Moore, C. A., Samayoa, J. A. G., Boggs, S. T., & Ladanyi, J. T. (2021). Social distancing decreases an individual’s likelihood of contracting COVID-19. Proceedings of the National Academy of Sciences, 118(8). DOI: 10.1073/pnas.2023131118 PMC7923674 33542156

[13] Fetzer, T., & Graeber, T. (2021). Measuring the scientific effectiveness of contact tracing: Evidence from a natural experiment. Proceedings of the National Academy of Sciences, 118(33). DOI: 10.1073/pnas.2100814118 PMC8380024 34385318

[14] Freeman, D., Lambe, S., Yu, L. M., Freeman, J., Chadwick, A., Vaccari, C., … & Loe, B. S. (2021). Injection fears and COVID-19 vaccine hesitancy. Psychological Medicine, 53(4), 1–11. DOI: 10.1017/S0033291721002609 34112276 PMC8220023

[15] Gelfand, M. J., Jackson, J. C., Pan, X., Nau, D., Pieper, D., Denison, E., … & Wang, M. (2021). The relationship between cultural tightness–looseness and COVID-19 cases and deaths: a global analysis. The Lancet Planetary Health, 5(3), e135–e144. DOI: 10.1016/S2542-5196(20)30301-6 33524310 PMC7946418

[16] Gelfand, M. J., Raver, J. L., Nishii, L., Leslie, L. M., Lun, J., Lim, B. C., … & Yamaguchi, S. (2011). Differences between tight and loose cultures: A 33-nation study. Science, 332(6033), 1100–1104. DOI: 10.1126/science.1197754 21617077

[17] Gollwitzer, P. M. (1999). Implementation intentions: strong effects of simple plans. American Psychologist, 54(7), 493–503. DOI: 10.1037/0003-066X.54.7.493

[18] Gollwitzer, P. M. (2012). Mindset theory of action phases. In P. A. Van Lange, A. W. Kruglanski, & E. T. Higgins (Eds.), Handbook of theories of social psychology (pp. 526–545). London: Sage. DOI: 10.4135/9781446249215.n26

[19] Gollwitzer, P. M., & Sheeran, P. (2006). Implementation intentions and goal achievement: A meta-analysis of effects and processes. Advances in Experimental Social Psychology, 38, 69–119. DOI: 10.1016/S0065-2601(06)38002-1

[20] Henrich, J., Heine, S. J., & Norenzayan, A. (2010). Most people are not WEIRD. Nature, 466, 29. DOI: 10.1038/466029a 20595995

[21] Hoerger, M. (2010). Participant dropout as a function of survey length in Internet-mediated university studies: Implications for study design and voluntary participation in psychological research. Cyberpsychology, Behavior, and Social Networking, 13(6), 697–700. DOI: 10.1089/cyber.2009.0445 21142995 PMC4367493

[22] Hofstede, G. (2011). Dimensionalizing cultures: The Hofstede model in context. Online Readings in Psychology and Culture, 2(1), 8. DOI: 10.9707/2307-0919.1014

[23] Howard, J., Huang, A., Li, Z., Tufekci, Z., Zdimal, V., van der Westhuizen, H. M., … & Rimoin, A. W. (2021). An evidence review of face masks against COVID-19. Proceedings of the National Academy of Sciences, 118(4). DOI: 10.1073/pnas.2014564118 PMC7848583 33431650

[24] Keller, L., Bieleke, M., & Gollwitzer, P. M. (2019). Mindset theory of action phases and if-then planning. In K. Sassenberg, & M. L. W. Vliek (Eds.), Social psychology in action (pp. 23–37). Cham: Springer. DOI: 10.1007/978-3-030-13788-5_2

[25] Keller, L., Gollwitzer, P. M., & Sheeran, P. (2020). Changing behavior using the model of action phases. In M. S. Hagger, L. Cameron, K. Hamilton, N. Hankonen, & T. Lintunen (Eds.), The handbook of behavior change (pp. 77–88). Cambridge: Cambridge University Press. DOI: 10.1017/9781108677318.006

[26] Knoll, M. D., & Wonodi, C. (2021). Oxford–AstraZeneca COVID-19 vaccine efficacy. The Lancet, 397(10269), 72–74. DOI: 10.1016/S0140-6736(20)32623-4 PMC7832220 33306990

[27] Kruglanski, A. W., Jasko, K., Chernikova, M., Milyavsky, M., Babush, M., Baldner, C., & Pierro, A. (2018). The rocky road from attitudes to behaviors: Charting the goal systemic course of actions. In A. W. Kruglanski (Ed.), The motivated mind: The selected works of Arie Kruglanski (pp. 253–298). New York: Routledge. DOI: 10.4324/9781315175867-7 26192134

[28] Lachowicz-Tabaczek, K., & Kozłowska, M. A. (2021). Being others-oriented during the pandemic: Individual differences in the sense of responsibility for collective health as a robust predictor of compliance with the COVID-19 containing measures. Personality and Individual Differences, 183, 111138. DOI: 10.1016/j.paid.2021.111138 34511682 PMC8416600

[29] Lammers, J., Crusius, J., & Gast, A. (2020). Correcting misperceptions of exponential coronavirus growth increases support for social distancing. Proceedings of the National Academy of Sciences, 117(28), 16264–16266. DOI: 10.1073/pnas.2006048117 PMC7368332 32581118

[30] Li, Y., Tenchov, R., Smoot, J., Liu, C., Watkins, S., & Zhou, Q. (2021). A comprehensive review of the global efforts on COVID-19 vaccine development. ACS Central Science, 7(4), 512–533. DOI: 10.1021/acscentsci.1c00120 34056083 PMC8029445

[31] Marinthe, G., Brown, G., Jaubert, T., & Chekroun, P. (2022). Do it for others! The role of family and national group social belongingness in engaging with COVID-19 preventive health behaviors. Journal of Experimental Social Psychology, 98, 104241. DOI: 10.1016/j.jesp.2021.104241 34690362 PMC8523484

[32] Mello, M. M., Opel, D. J., Benjamin, R. M., Callaghan, T., DiResta, R., Elharake, J. A., … & Caplan, A. (2022). Effectiveness of vaccination mandates in improving uptake of COVID-19 vaccines in the USA. The Lancet, 400(10351), 535–538. DOI: 10.1016/S0140-6736(22)00875-3 PMC9270060 35817078

[33] Milkman, K. L., Gromet, D., Ho, H., Kay, J. S., Lee, T. W., Pandiloski, P., … & Duckworth, A. L. (2021). Megastudies improve the impact of applied behavioural science. Nature, 600, 478–483. DOI: 10.1038/s41586-021-04128-4 34880497 PMC8822539

[34] Morabia, A. (2023). Republicans die more from COVID-19: Why we care. American Journal of Public Health, 113(4), 349–349. DOI: 10.2105/AJPH.2023.307237 36888943 PMC10003493

[35] Mukherjee, S., Vincent, C. K., Jayasekera, H. W., & Yekhe, A. S. (2021). Antiviral efficacy of personal care formulations against Severe Acute Respiratory Syndrome Coronavirus 2. Infection, Disease & Health, 26(1), 63–66. DOI: 10.1016/j.idh.2020.09.003 PMC7498208 33012695

[36] Obrecht, N. A., & Chesney, D. L. (2016). Prompting deliberation increases base-rate use. Judgment & Decision Making, 11(1), 1–6. DOI: 10.1017/S1930297500007543

[37] Papa, A., & Follette, W. C. (2015). Dismantling studies of psychotherapy. In R. L. Cautin, & S. O. Lilienfeld (Eds.), The encyclopedia of clinical psychology (pp. 1–6). DOI: 10.1002/9781118625392.wbecp523

[38] Peer, E., Rothschild, D., Gordon, A., Evernden, Z., & Damer, E. (2022). Data quality of platforms and panels for online behavioral research. Behavior Research Methods, 54, 1643–1662. DOI: 10.3758/s13428-021-01694-3 34590289 PMC8480459

[39] Puca, R. M., & Schmalt, H. D. (2001). The influence of the achievement motive on spontaneous thoughts in pre-and postdecisional action phases. Personality and Social Psychology Bulletin, 27(3), 302–308. DOI: 10.1177/0146167201273004

[40] Robinson, S. A., Bisson, A. N., Hughes, M. L., Ebert, J., & Lachman, M. E. (2019). Time for change: using implementation intentions to promote physical activity in a randomised pilot trial. Psychology & Health, 34(2), 232–254. DOI: 10.1080/08870446.2018.1539487 30596272 PMC6440859

[41] Schmitz, M., Wollast, R., Bigot, A., & Luminet, O. (2022). Predicting health behaviors across Belgium and France during the first wave of COVID-19 pandemic. Journal of Health Psychology, 27(14), 3097–3105. DOI: 10.1177/13591053221083819 35297292 PMC9720059

[42] Schultz, P. W., Nolan, J. M., Cialdini, R. B., Goldstein, N. J., & Griskevicius, V. (2018). The constructive, destructive, and reconstructive power of social norms: Reprise. Perspectives on Psychological Science, 13(2), 249–254. DOI: 10.1177/1745691617693325 29592653

[43] Sheeran, P., Maki, A., Montanaro, E., Avishai-Yitshak, A., Bryan, A., Klein, W. M., … & Rothman, A. J. (2016). The impact of changing attitudes, norms, and self-efficacy on health-related intentions and behavior: A meta-analysis. Health Psychology, 35(11), 1178–1188. DOI: 10.1037/hea0000387 27280365

[44] Sheeran, P., & Webb, T. L. (2016). The intention–behavior gap. Social and Personality Psychology Compass, 10(9), 503–518. DOI: 10.1111/spc3.12265

[45] Silva, M. A. V. D., Sao-Joao, T. M., Brizon, V. C., Franco, D. H., & Mialhe, F. L. (2018). Impact of implementation intentions on physical activity practice in adults: A systematic review and meta-analysis of randomized clinical trials. PLoS One, 13(11), e0206294. DOI: 10.1371/journal.pone.0206294 30427874 PMC6235272

[46] Talic, S., Shah, S., Wild, H., Gasevic, D., Maharaj, A., Ademi, Z., … & Ilic, D. (2021). Effectiveness of public health measures in reducing the incidence of covid-19, SARS-CoV-2 transmission, and covid-19 mortality: systematic review and meta-analysis. BMJ, 375, e068302. DOI: 10.1136/jech-2020-215256 34789505 PMC9423125

[47] Van Bavel, J. J., Cichocka, A., Capraro, V., Sjåstad, H., Nezlek, J. B., Pavlović, T., … & Jørgensen, F. J. (2022). National identity predicts public health support during a global pandemic. Nature Communications, 13(1), 1–14. DOI: 10.1038/s41467-021-27668-9 PMC8792004 35082277

[48] Van Bavel, J. J. V., Baicker, K., Boggio, P. S., Capraro, V., Cichocka, A., Cikara, M., … & Willer, R. (2020). Using social and behavioural science to support COVID-19 pandemic response. Nature Human Behaviour, 4(5), 460–471. DOI: 10.1038/s41562-020-0884-z 32355299

[49] Wang, Y. (2021). Government policies, national culture and social distancing during the first wave of the COVID-19 pandemic: International evidence. Safety Science, 135, 105138. DOI: 10.1016/j.ssci.2020.105138 36570788 PMC9759369

[50] Ward, J. K., Gauna, F., Gagneux-Brunon, A., Botelho-Nevers, E., Cracowski, J. L., Khouri, C., … & Peretti-Watel, P. (2022). The French health pass holds lessons for mandatory COVID-19 vaccination. Nature Medicine, 28, 232–235. DOI: 10.1038/s41591-021-01661-7 35022575

[51] Wollast, R., Schmitz, M., Bigot, A., & Luminet, O. (2021). The theory of planned behavior during the COVID-19 pandemic: A comparison of health behaviors between Belgian and French residents. PloS One, 16(11), e0258320. DOI: 10.1371/journal.pone.0258320 34735473 PMC8568184

